# 

**Published:** 1918-05-11

**Authors:** 


RATES OF SUBSCRIPTION: Twelve months, 6/6; Six months, 4/-; three months, 2/-, post free to any address in the United Kingdom. Abroad, Twelve months, 8/8; Six months, 5/-; Three months, 2/6, post free. **THE HOSPITAL "Index" to Volume LXIII, October 1917 to March 1918, is now ready, price 3½d. per copy, post free.** Address The Manager, THE HOSPITAL, "The Hospital" Building, 28/29 Southampton Street, Strand, London W.C. 2.

St. Thomas's Hospital House Appointments.—The following have been selected from April 30, 1918: *Casualty Officers and Resident Anæsthetists.*—C. H. Vernon, E. J. S. Bonnett, M.R.C.S., L.R.C.P.; C. E. Cobb, N. B. de. M. Greenstreet. *Resident House Physicians.*—T. Patterson, A. T. Spoor. *Resident House Surgeons.*—F. C. Alton, F. G. Hobson, E. N. Showell-Rogers. *House Surgeon to Block* 8.—T. M. Dilworth. *Obstetric House Physician.*—H. B. Russell. *Clinical Assistants.*—(*Throat*) R. A. Walker, (*Ear*) H. I. Marriner, (*Children's Surgical*) G. Murray Levick, (*Electrical and X-ray Department*) A. R. Hargreaves.

### Forthcoming Events.

Inter-Allied Conference on the After-Care of Discharged Sailors and Soldiers.—Central Hall, Westminster, May 20 to May 25. Opening ceremony by H.R.H. the Duke of Connaught, 11.30 A.M., Monday, May 20.

Theatrical Garden Party.—In aid of the Actors' Orphanage, Tuesday, June 25, at the Botanic Gardens, Regent's Park.

Queen's Hospital for Children, Hackney Road, E. 2.—Annual Meeting of the Governors in the Board Room, on Monday, 13th inst., at 4 P.M.

Central Council for District Nursing in London.—Conference on Maternity Nursing, in the Board Room of the Metropolitan Asylums Board, Embankment, E.C., Tuesday, June 4, 5 P.M., Sir William J. Collins, M.P., M.D., K.C.V.O., in the chair.

Nurses' National Total Abstinence League.—A meeting will be held in the Out-Patient Hall of the London Temperance Hospital, Hampstead Road, N.W. (by kind permission of the Board of Management), on Saturday, May 11, at 3.30 P.M. Dr. Catherine Ironside and Captain Arthur Evans, F.R.C.S., will speak.

### R.A.M.C. Travelling Expenses.

SIR H. CRAIK asked the Financial Secretary to the War Office whether medical men who are called up to serve as officers in the Royal Army Medical Corps are required to pay their own railway fares from their homes to London. Mr. Forster replied that officers do not necessarily join for duty in London on first appointment, but proceed from their homes to their stations direct. Free travelling for this journey is not admissible.

### THE COLLEGE OF NURSING (Limited by Guarantee).

### Booming Ahead!

### THE THIRD ANNUAL REPORT.

The presentation of the Third Annual Report finds the College at a most interesting and hopeful stage of its development, and although, owing to conditions which it was impossible to anticipate, progress has been somewhat retarded, the advance already achieved most assuredly justifies the encouragement and confidence felt by the Council in entering upon a new year.

2. When last year's Report was presented the strength of the Register stood at 2,553, and it is with pleasure that the Council is able to state that at the end of the financial year, March 31, 1918, the Membership of the College stood at 7,962 Trained Nurses, and at the date of this Report numbers 8,263. It is a matter for congratulation that the personal effort made by these Nurses is a substantial proof of their desire to contribute to the organisation of their Profession. A large number even of Nurses engaged upon Active Service have registered during the past year, and the increased personal interest shown in the developments of the College has been well marked.

3. The Council expresses its gratitude to the Nursing Press for the support it continues to give the College in keeping its activities before the Nurses. Such help, always welcome, is invaluable at this stage of its work. Similar support has also been extended to its efforts by the general Press, and much assistance has been afforded by the circulation of a pamphlet entitled "A Nurse's Talk to Nurses," written by Miss V. Matheson, Secretary of the Irish Board, which states clearly the aims and objects of the College of Nursing.

4. The Council has to record with regret the resignation from the Council, through illness, of Miss Haughton, R.R.C., late Matron of Guy's Hospital. Miss Haughton was one of the first Matrons to welcome the establishment of a College of Nursing, and much of the initial success of the College is due to her efforts. Miss Haughton's retirement is felt by the Council to be a great loss.

5. The Council also reports with regret the death of five Members during the year, two of whom are on the Roll of Honour—viz., Miss Agnes Murdock Climie, died on Active Service, October 1917; and Miss Charlotte Edith Henry, drowned, March 1918.

6. Meetings have been held in various parts of the country in support of the College, and as the result of this propaganda Local Centres have already been formed in Liverpool, Manchester, Birmingham, Bristol, and Leicester, while others are in process of formation elsewhere. The Local Representatives of these Centres are as follows:—*Birmingham.*—Miss A. M. Bodley, Selly Oak Infirmary, assisted by Miss Blantyre Shaw. *Bristol.*—Hon. Sec., Miss G. E. N. White, Sister T.F.N.S., 2nd Southern General Hospital, Bristol; Miss McHardy, Matron, Chesterfield Place, Nursing Home, Bristol. *Leicester.*—Miss Masters, Matron, Poor-Law Union Infirmary. *Liverpool.*—Miss C. Worsley, Matron, Infirmary for Children, Liverpool. *Manchester.*—Miss Maude Smith, R.R.C., South Manchester Hospitals; Hon. Secs., Miss Earl, Matron, Ancoats Hospital; Miss Pilgrim, Inspector, Queen's Nursing, Cheshire and Lancashire.

The lively enthusiasm of these Local Centres is manifested in the varied character of the inquiries upon which information and leading are sought. These Centres having been formed as a medium for developing the ideals of the Profession, their educational character will, the Council hopes, be jealously guarded. It is pleasing to record that courses of post-graduate lectures in connection with the Manchester, Liverpool, and Leicester Branches are already announced.

7. It will be readily understood that the College has a stupendous task in front of it, and a task it will be impossible to achieve without the aid of a substantial endowment. Realising this necessity, approaches were sanctioned by the Council to the British Women's Hospital Committee, who in the early part of the War had raised the Star and Garter Fund for Disabled Soldiers, and assisted largely the work of the Scottish Women's Hospital, with a view to raising a Central Fund which should be for the endowment of the College, and a provision for those women who had broken down in health. The Government has made a certain provision for Nurses engaged in the Naval and Military Services, but there are thousands of Nurses who at the present moment are safeguarding the health of the Nation at home, and the burden consequent on their depleted numbers is increasingly heavy. Our gratitude is due to the British Women's Hospital Committee, who consented to issue this appeal as a thankoffering of the British Empire to British Nurses. This fund it is hoped will so establish the organisation of the Nursing Profession that it shall in the future be in a sound economic position. We feel sure that the Nurses will make individual effort to support it in every way possible.

8. The Council records with much satisfaction the support given to the College by the various Nurse Training Schools, and the efforts made to bring the standard of training up to the requirement of the College. At the invitation of different hospitals, members of the Council have inspected the conditions of training with a view to the recognition of their Certificates as a qualification for Membership of the College during the period of grace. The Consultative Committee, appointed by the Council to consider the curricula of Training Schools, has availed itself of its powers to enlarge its representative nature by inviting Members of the Profession outside the College Council to meet them in conference. This additional representation has proved of great assistance to the Committee, and details of curricula are now under discussion. As a preliminary step the assistance of the Training Schools has been sought as to the prevailing conditions of training at the present date.

9. The negotiations to which allusion was made in last year's Report, entered into at the beginning of the year 1916, between the College and the Royal British Nurses' Association, with a view to the amalgamation of the two Bodies, were continued until September 1917. Unfortunately, the alterations made by the Privy Council in the Supplemental Charter, though readily accepted by the College as not in any material degree modifying the sense of that document, were regarded as unacceptable by the Council of the Royal British Nurses' Association, and thus a movement which at one time was full of promise did not reach fulfilment. The constructive work of the College, including the presentation to Parliament of a Bill to provide for the State Registration of Nurses, has in consequence been retarded, since, during the period of negotiations with the Royal British Nurses' Association, the Council was naturally unwilling to anticipate the precise terms of a Bill which in the event of an amalgamation it would have been the duty of the Council of the Amalgamated Bodies to formulate in detail.

10. Members will naturally be interested to learn what the course of negotiations has been with the Central Committee with a view of obtaining an agreed Bill to present to Parliament for the training and registration of Nurses. It will be remembered that they were broken off by the Central Committee in July 1916, the principal point at issue being the setting out in the Bill of the names of the various bodies, including the organised Societies of Nurses, which should nominate the Provisional Council. At the beginning of last July Sir Arthur Stanley informed Major Chapple, who is in Parliamentary charge of the Central Committee's Bill, that the Council was prepared to concede this point, and it was therefore hoped that the Central Committee would reopen negotiations. This, however, has not been done, but lately Sir Arthur Stanley and Major Chapple have been in communication with each other, so as to arrive if

### THE COLLEGE OF NURSING—(*continued*).

possible at a Bill which, agreed between themselves, will be recommended for acceptance by the Council of the College and the Central Committee for the State Registration of Nurses respectively. Members may rely upon the Council to give very careful scrutiny to the terms of the Bill, and in particular to the essential points of safeguarding the position of the Members of the College upon the Legal Register, and of setting out that not fewer than two-thirds of its General Nursing Council shall be elected by the Nurses on the Register.

11. Certain Members of the College having drawn the attention of the Council to the bestowal of the Military Medal upon Members of the Nursing Profession engaged on Active Service, pointing out that this award is a decoration confined to non-commissioned officers and men, and that the appropriate decoration for the Army Nursing Service is the Military Cross bestowed on Officers, a letter was written to the Secretary of State for War to that effect. A reply was received from the Army Council stating that "the Royal Red Cross conferred on a Nurse, in recognition of her Professional Services as a Nurse, is to be considered as equivalent to the Military Cross awarded to an Officer for Professional Services in the Field. Moreover, as the Military Medal can be awarded to both men and women, when conferred upon a Nurse for bravery in the Field such award is for bravery apart from her professional duties, and is, therefore, won in her capacity as a woman, not necessarily because she is a Nurse." The Council of the College proposes at an early date to re-open the matter with the Military Authorities.

12. At the request of many Members of the College, a Badge Committee has been formed to consider designs which have been submitted for the approval of the Council, but no decision has yet been reached.

13. From this Report it will be seen that the progress made during the past year is substantial, and that the College is still advancing and strengthening itself, not only in numbers, but in financial reserves. Moreover, the increasing support and interest it receives from Members of the Profession justifies the confident hope that it will realise all that was anticipated, and become a medium through which the Nurses will be able themselves to organise their own affairs. An earnest of this is afforded by the interest already shown by the Members in the nomination of Candidates for the vacancies on the Council, and on the first occasion on which so large an electorate has been appealed to, every Member should feel it incumbent on her to record her vote. Thus when it enters upon the task that awaits it in the coming year the Council will feel its hands considerably strengthened by the fact that one-third of its number has been elected directly by the Nurses on the Register.

By Order of the Council,

Arthur Stanley, Chairman.

April 18, 1918. Mary S. Rundle, Secretary.

### THE ANNUAL REPORT OF THE SCOTTISH BOARD.

During the past year the Scottish Board has met four times, the Registration Committee eleven times, and the General Purposes Committee five times, the meetings being held alternately in Edinburgh and Glasgow.

The Board regrets to record the loss of one of its members, Miss Peterkin, who, having been appointed General Superintendent of the Q.V.J.I.N. in London, was obliged to resign; they feel assured that Miss Peterkin's interest in the work will continue, and they have greatly appreciated the valuable services she rendered on the Board. Miss Rumsey, who succeeded Miss Peterkin as General Superintendent of the Scottish Branch of Queen Victoria's Jubilee Institute for Nurses, has kindly consented to replace her on the Board. The Board has also to welcome another new member, Miss MacCalman, District Nurse, Perth, who has been appointed to fill the remaining vacancy for a Nurse member.

The work in Scotland has progressed steadily, the total number of Scottish Members of the College on March 31 being 831. During the year 606 applications have been considered; of these, 550 have been recommended to the Council of the College for admission to the Register, 46 have been rejected, 1 has withdrawn, and 9 are still under consideration.

Some of the smaller hospitals have invited inspection on behalf of the College. Two representatives of the Board, Dr. Claude Ker and Miss Gill, have inspected two of these hospitals and furnished reports with recommendations. Other hospitals are awaiting inspection, and will be visited as soon as the necessary arrangements can be made.

Propaganda work has been carried on in such large centres as Edinburgh, Glasgow, and Aberdeen. The Secretary has organised meetings in the various towns, and she has been invited to speak in some of the principal hospitals. In her efforts to explain the work she has been helped by several members of the Board, who have attended the meetings and spoken on the aims of the College.

*Finance.*—The sum of £243 3s. 5d. was received from the Hon. Treasurer of the College of Nursing for the year March 1917-1918, and the cash balance in hand on March 31, 1917, was £171 7s. 4d. The expenditure for the year has been £355 13s. 10d., as shown in the Balance Sheet, and the cash balance in hand on March 31, 1918, was £60 11s. 11d.

Miss A. W. Gill, R.R.C., Lady Superintendent, Royal Infirmary, Edinburgh, Hon. Treasurer and Hon. Secretary.

James Ritchie, Chairman.

Mary A. Brunton, Secretary.

### THE ANNUAL REPORT OF THE IRISH BOARD.

When the last Annual Report of the College was issued it included a brief communication from the Irish Branch, which had then been in existence for about four months. Since that time the College in Ireland has made steady, if slow, progress, and though no startling events have taken place, and there has been no sudden flocking of nurses to join the College, the progress which the Board is able to report is as satisfactory as could be expected under the difficult circumstances in which the Board is placed.

Since last June there has been a steady monthly average of between 20 and 30 applications for Registration, and the number of Members trained in Ireland who have paid their Registration Fee now amounts to 268. Besides these, there are some 160 nurses in Ireland who have joined the College, but who received their training in England. The Members are drawn from all parts of the country, Belfast and the Ulster Counties leading the van, while Limerick has done particularly well, and the number of nurses coming from the County Clare is also encouraging.

Meetings in support of the College have been held in Dublin, Belfast, and Cork, and the Board has endeavoured to make its aims and objects known throughout the country. Owing to the existence of a rival organisation this task has been particularly difficult, and there remains a great deal of confusion in the minds of nurses, which patience and perseverance alone will dissipate. The Board most sincerely deprecate the emphasis which the establishment of a rival institution has laid upon existing differences in a country where every effort towards unity is to be eagerly desired and welcomed, and where the Nursing Profession is in especial need of organisation and encouragement, and the elevation of status which co-operative effort alone can achieve.

In view of the generally accepted dictum that the economic condition of the Nursing Profession in this country is deplorable, the Board has endeavoured to collect statistics as to the cost of hospital training to the nurse, and the average salaries received in the different nursing positions. Owing to the bitterness of feeling amongst those opposed to the College it has not been possible to obtain all the

### THE COLLEGE OF NURSING—(*continued*).

information desired, but the figures received from some 60 institutions show that the economic status of the nurse in this country is not only inferior to that of nurses in England, but that it is disproportionately so, even when the poverty of Ireland as compared with England is taken into consideration. The improvement of these conditions is a matter which cannot be effected in the twinkling of an eye, but the Board are hopeful that organisation and persevering endeavour will enlighten public opinion and bring about much-needed reforms.

The Irish Branch hopes to make a small beginning of practical benefit to its Members in the coming autumn, by establishing a Reading Room in connection with its office, where they can meet their friends, read, write letters, and get tea. It is hoped that this may be the beginning of a keener and more vigorous interest of Members in the affairs of their own Profession.

In connection with the Nation's Fund for Nurses, Ireland has not yet embarked upon any definite work, but it is hoped that before very long she will do her share in contributing to the endowment of the College and the establishment of the much-needed Benevolent Fund.

The annual election of the Board took place in March, when six of the original Members retired. There were already two vacancies on the Board, making eight in all. Thirteen nominations were received, and the voting, which was by postal ballot, resulted in the election of the following Members, of whom four are re-elected: Miss Bostock, Matron, Royal Victoria Hospital, Belfast; Miss Chisholm, Act. Supt. Irish Branch Q.V.J.I.N.; Miss Agnes Adams, Sister, Adelaide Hospital, Dublin; Miss E. J. Richardson, Sister, Royal City of Dublin Hospital; Dr. Ella Webb, Dublin; Charles Benson, Esq., M.D., F.R.C.S.I., Dublin; Sir Alexander Dempsey, M.D., F.R.C.S.I., Belfast; Thomas M. Tate, Esq., M.D., Down County Infirmary, Downpatrick.

In conclusion, the Board are of opinion that the establishment of an Irish Branch of the College has been thoroughly worth while. It has been the means of arousing interest in the nursing question in Ireland, of bringing together those who have been doing their utmost as individuals to promote the welfare of nurses, and of quickening into activity the slumbering indifference which has for so many years allowed the Profession as a whole to drift along the line of least resistance. That much of this activity should be manifested in hostility to the College is regrettable, but the Irish Board are confident that by patient and persistent endeavour the aims of the College will ultimately be realised in this country.

Andrew Beattie, Esq., D.L., Hon. Treasurer; Miss Reid, Ivanhoe Nursing Home, Dublin, Hon. Secretary.

(Signed) George Peacocke, Chairman.

### THIRD ANNUAL REPORT OF THE HON. TREASURER.

During the present financial year Miss Sydney Browne, R.R.C., Matron-in-Chief of the Territorial Force Nursing Service, and the Hon. Sir William Goschen, K.B.E., have been elected co-Honorary Treasurers with Mr. Comyns Berkeley, from April 1, 1918.

The audited accounts as presented cover the period from April 1, 1917, to March 31, 1918, during which time 5,414 Members were registered and paid their fees, as against 2,553 in the previous year.

The surplus on the Income and Expenditure Account for the year is £4,998 13s. 2d., which sum has been added to the Accumulated Fund of the College. This balance is subject to a reduction with respect to the expenses incurred in connection with the proposed amalgamation of the College with the Royal British Nurses' Association. An account of these, however, has not yet been rendered; this liability, therefore, cannot at present be stated.

The audited accounts of the Scottish and Irish Boards are submitted. Grants have been made during the year to these Boards. The Scottish Board, having a substantial balance brought forward on March 31, 1917, was not in need of funds to the same extent as the Irish Board, which was only established about four months before the commencement of the present financial year. It is interesting to note that whereas up to March 31, 1917, only 185 Members were registered from Scotland, since that date 646 Members have been registered and have paid their fees, and 230 more Irish Members have been registered during the same period.

It was decided by the Council of the College to transfer the General Purposes Fund to the Accumulated Fund, which now totals £8,692 10s. 10d., until the time arrives for the erection of a building for the purposes of the College. The Accumulated Fund has been invested mostly in 5 per cent. War Loan and National War Bonds.

**When the Council decided upon the sum of £1 1s. as the fee to be paid by a Nurse on registration as a Member of the College, it did so with the intention that the odd shilling should be used towards the expenses entailed in the registration, such as postage, stationery, etc. It is very satisfactory to note, however, that for the present financial year the amount of the Accumulated Fund is more than the total amount of fees received from the Members of the College for registration, inclusive of all expenses of management to date, these having been met by donations and the interest on investments.**

The accounts do not include any money contributed by the Nation's Fund for Nurses.

The Council again offers its best thanks to Sir James Boyton, M.P., for the loan of two rooms at No. 6 Vere Street, free of all rent, rates, lighting, and heating.

The financial position of the College is most satisfactory.

(Signed) Comyns Berkeley, Hon. Treasurer.

March 31, 1918.

A Notice of the Report will be found on page 123.

